# Healthcare professionals’ perspectives on artificial intelligence in clinical practice: a systematic review of facilitators and challenges

**DOI:** 10.1093/oodh/oqag004

**Published:** 2026-02-10

**Authors:** Gowtham Pallamala, Judy Jenkins, Panamparampil Kurian Sherin, Ashly Nyathi, Jomin George

**Affiliations:** Department of Health Informatics, Swansea University Medical School, Swansea University, Swansea, United Kingdom; Department of Health Informatics, Swansea University, Singleton Park, Swansea, Wales SA2 8PP, United Kingdom; Shri K LShastri Smarak Nursing College, Lucknow, Uttar Pradesh, India; Midlands State University, Senga, Gweru, Zimbabwe; School of Nursing and Midwifery, Royal College of Surgeons, Dublin, Ireland

**Keywords:** artificial intelligence (AI), machine learning and deep learning, clinical decision support systems (CDSS), healthcare professional perspectives, ethics, safety and legal issues

## Abstract

Artificial intelligence (AI) tools enhance health care by decision making, reducing errors, and delivering the best care to patients. Healthcare professionals are the users of the AI tools, and it is essential to have knowledge and skills in the utilization of AI tools to deliver effective care. Therefore, this study systematically explores the healthcare professional perspectives in using AI tools in clinical practice and identifies the facilitators and challenges associated with their usage. Three databases, PubMed, EBSCOhost, and ACM Digital Library, are systematically used to identify qualitative research studies. Appropriate selection of research articles is carried out by the inclusion and exclusion criteria. The Critical Appraisal Studies Programme tool is utilized for assessment. Data are extracted and analysed effectively. Out of 1292 articles,10 qualitative research studies which meet the objectives of the research are included. By analysing the information, six themes developed are behaviour, perceived usefulness, performance expectancy, ethical and legal aspects, challenges, and AI tool proficiency. Healthcare professionals acknowledge the value of AI tools in clinical practice; however, clinicians often lack the necessary competencies for their effective deployment. It is therefore imperative that healthcare practitioners collaborate with developers during the design phase of AI systems, ensuring due consideration of ethical and legal requirements. While AI technologies offer numerous advantages, prioritizing transparency and explainability is essential to optimize their integration within clinical workflows. Ongoing proficiency with AI tools may be sustained through structured training programmes and the establishment of clear operational guidelines.

Research in context **Evidence before this study**

PubMed, EBSCOhost, and the ACM Digital Library (January–March 2025) were searched for primary qualitative studies (2018–23) using combinations of ‘artificial intelligence’, ‘healthcare professionals’, ‘clinical practice’, ‘perspectives’, ‘facilitators’, and ‘barriers’. While numerous trials and reviews have evaluated AI’s diagnostic accuracy and health-economic impact, no prior review has synthesized healthcare professionals’ own experiences of using AI tools in everyday clinical work or identified the factors that support or impede their adoption.

**Added value of this study**

This systematic review is the first to collate and thematically analyse healthcare professionals’ perspectives on AI tools across varied clinical settings. Key facilitators comprised hands-on training, early involvement of end-users in system design, and transparent algorithm explainability; principal challenges included skills deficits, concerns over legal and ethical accountability, and poor integration with existing workflows. By foregrounding clinicians’ lived experiences, we provide a nuanced account of what enables and what hinders effective AI implementation.

**Implications of all the available evidence**

Our findings highlight that embedding structured training programmes, formal guidelines on ethics and liability, and ongoing collaboration between developers and frontline staff are essential to foster trust and optimize AI’s clinical utility. Policymakers, educators, and healthcare organizations should prioritize these measures to ensure safe, equitable, and sustainable adoption of AI technologies in routine patient care.

## Introduction

Artificial intelligence (AI), as defined by Russell et al. (2023), comprises ‘computer science techniques that mimic human intelligence, including algorithms that leverage machine learning, deep learning, natural language processing, and neural networks’ (p. 348). These technologies transform extensive and complex datasets into actionable insights, aiding clinical decision-making (Ganapathi and Duggal 2023). The adoption of AI in healthcare is expanding rapidly, demonstrating significant benefits in areas such as the diagnosis of ambiguous skin lesions (Haugsten et al. 2023), AI-supported decision-support systems(Samhammer et al. 2022), early detection of diabetic retinopathy (Held et al. 2022), prompt identification of heart failure (Barry et al. 2022), and in optimizing antibiotic prescribing practices (Huang et al. 2023). Primary care presents unique challenges for AI integration, due to its broad clinical scope, diverse patient populations, and varying health outcomes (Nash et al. 2023). Moreover, machine- and deep-learning techniques have demonstrated enhanced accuracy in cancer diagnosis, occasionally surpassing expert clinicians’ performance (Samhammer et al. 2022). However, AI application in obstetrics and gynaecology remains nascent, particularly regarding pregnancy-related decision-making, where tools are still under development (Fischer et al. 2023).

The principal aims of incorporating AI into healthcare include improving quality of care, minimizing expenditure, and promoting public health across patient, organizational, and professional domains (Ganapathi and Duggal 2023). Nonetheless, frontline healthcare practitioners must individually overcome implementation challenges (Barry et al. 2022), thereby necessitating specific competencies for the provision of safe and effective AI-supported care (Russell et al. 2023). Since its emergence, AI has prompted ethical, legal, and practical concerns, alongside developmental inaccuracies which may perpetuate healthcare inequalities (Russell et al. 2023). It is, therefore, essential to examine barriers, enablers, safety issues, and ethical considerations to sustain trust among clinicians and patients in AI systems (Held et al. 2022).

Healthcare professionals’ insights are fundamental in designing AI solutions, given their position as primary users and informants of clinical needs (Fischer et al. 2023). Understanding clinicians’ skills requirements and perspectives on trust, accuracy, ethics, legal responsibility, and the patient–physician relationship is crucial for ensuring the safe integration of AI technologies in clinical practice (Samhammer et al. 2022, Fischer et al. 2023). This study consequently seeks to explore these perceptions and the corresponding facilitators and barriers to AI adoption in healthcare.

## Research question

What are the perspectives of healthcare professionals using AI tools in clinical practice, and what facilitators and challenges are associated with their use?

## Aim

The aim of the study is to explore the perspectives of healthcare professionals in using AI tools in clinical practice and to identify the facilitators and challenges associated with their use.

## Objectives

1. To identify the factors that support and hinder the use of AI tools in clinical practice, as experienced by healthcare professionals.
2. To explore the perspectives of healthcare professionals regarding the utilization of AI-based tools.

## Rationale

The global rise in chronic diseases and the emergence of advanced treatment methods have led to increased healthcare costs (Higgins et al. 2020). AI offers a promising, cost-effective means to improve healthcare delivery (Higgins et al. 2020). The integration of AI into clinical decision support systems enhances diagnostic accuracy and clinical decision-making (Amann et al. 2020). AI technologies support a broad spectrum of essential services and contribute to poverty reduction by expanding healthcare access in resource-limited areas (Wahl et al. 2018). From an economic standpoint, AI fosters development by lowering healthcare expenditure (Wahl et al. 2018). For AI tools to be effective in clinical settings, the perspectives of clinicians—as primary users—are crucial (Lai et al. 2020). Their insights are indispensable in aligning AI development with practical needs (Ross et al. 2016). Addressing the existing literature gap on healthcare professionals’ views is vital for the successful implementation of AI (Lai et al. 2020, Fischer et al. 2023).

## Materials and methods

### Research design

In this study, a qualitative systematic review was carried out to identify knowledge gaps and develop guidelines for future research. A qualitative approach enables exploration of healthcare providers’ perspectives, expectations, and concerns regarding the use of AI tools through open-ended enquiries, thereby elucidating end-user requirements in real-world clinical settings. This study employed established qualitative research techniques to address both the ‘how’ and ‘why’ questions and to capture the multifaceted nature of participants’ experiences rather than merely numerical data (Cleland et al. 2017).

### Ethics statement

This systematic review utilized publicly available, de-identified data; therefore, ethical approval was not required.

### Role of the funding source

This research received no specific grant from any funding agency in the public, commercial or not-for-profit sectors in study design, data collection, data analysis, data interpretation or writing of the report.

### Search strategy

Various databases—including PubMed, EBSCOhost and the ACM Digital Library—were searched for primary research papers (Eriksen and Frandsen 2018). These peer-reviewed sources were selected for their reliability and full-text availability. We included qualitative studies published between 2018 and 2023. Boolean operators ‘AND’ and ‘OR’ were used to refine search precision by narrowing or expanding the coverage of retrieved articles (Bramer et al. 2018). While the review was restricted to PubMed, EBSCOhost, and ACM Digital Library, additional relevant studies from human computer interaction provide complementary insights into how clinicians negotiate, accept, or reject recommendations. These include (Yang et al. 2019, Sendak et al. 2020, Henry et al. 2022, Sivaraman et al. 2023). Future studies should interpret both medical and Human Computer Interaction (HCI) perspective for more holistic views.

### SPIDER

**Sample**: Healthcare professionals, including nurses, physicians and specialists.

**Phenomenon of Interest**: Use of AI tools (machine learning, deep learning, natural language processing, and neural networks).

**Design**: Qualitative systematic review exploring healthcare professional perspectives from 2018 to 2023.

**Evaluation**: Primary research studies reporting healthcare professionals’ experiences with AI tools in clinical practice, and the facilitators and barriers to their use.

**Research Type**: Qualitative studies employing semi-structured interviews and focus groups to explore healthcare professionals’ perspectives.

### Search terms

‘Artificial Intelligence’, ‘healthcare’, ‘decision making’, ‘experience’, ‘healthcare professionals’, ‘treatment’, ‘diagnosis’, ‘machine learning’, ‘perception’, ‘clinical decision support system’, ‘antibiotic prescribing’, ‘qualitative research’, ‘informatics’, ‘family medicine’, ‘obstetrics’, ‘gynaecology’, ‘physician’, ‘health personnel’, ‘AI deployment’, ‘rural clinics’, ‘human–AI collaboration’, ‘trust in AI’, ‘CDSS’, ‘computer-assisted diagnosis’, ‘dermatology’, ‘diagnostic tool’, ‘curriculum’, ‘educational status’, ‘primary care’, ‘screening’, ‘eHealth’, ‘diabetic retinopathy’, ‘AI-enabled clinical decision support’, ‘human–computer interaction’, ‘health care delivery’, ‘health informatics’, ‘cardiology’, ‘AI tools’.

### Selection criteria

An appropriate selection process was employed using the predefined inclusion and exclusion criteria as shown in Table 1. Qualitative research studies exploring healthcare professionals’ perspectives on AI tools and AI-supported applications in clinical practice were selected. Qualitative studies involving semi-structured and focus-group interviews, which provided information on healthcare providers’ knowledge, facilitators, concerns, and requirements, were included. Primary, qualitative, peer-reviewed, full-text articles published in English between 2018 and 2023 were eligible for inclusion.

**Table 1:** Inclusion and exclusion criteria.

Inclusion criteria	Exclusion criteria
Healthcare professionals like physicians, pharmacists, nurses, social workers of healthcare, physicians of various professions like radiology, cardiology, diabetes, nephrology, pathology, pulmonology. It includes varied experience.	Non-health professionals, patients, and non-medical professionals.
Artificial intelligence tools in clinical practice, AI-based clinical decision support system, machine learning, deep learning, algorithm-driven decisions, precision medicine, computer simulation, natural language processing, neural networks, decision making.	Studies which are not associated with AI are excluded.
Primary qualitative studies obtain healthcare professional perspectives.	Secondary research studies, quantitative studies, and articles not relating to health professional perspectives.
Peer-reviewed articles were included in the study.	Grey literature, opinion articles, blogs, and websites.
Articles published in the English language only.	Articles not in English.
Year of publication from 2018 to 2023.	Published before 2018.

### PRISMA

The Preferred Reporting Items for Systematic Reviews and Meta-Analyses (PRISMA) statement provides guidelines for reporting systematic reviews in the healthcare sector (Page et al. 2021). PRISMA outlines the flow of information across the various stages of the review process, and comprehensive reporting enables readers to appraise the appropriateness of the methods used (Page et al. 2021).

**Figure 1:**
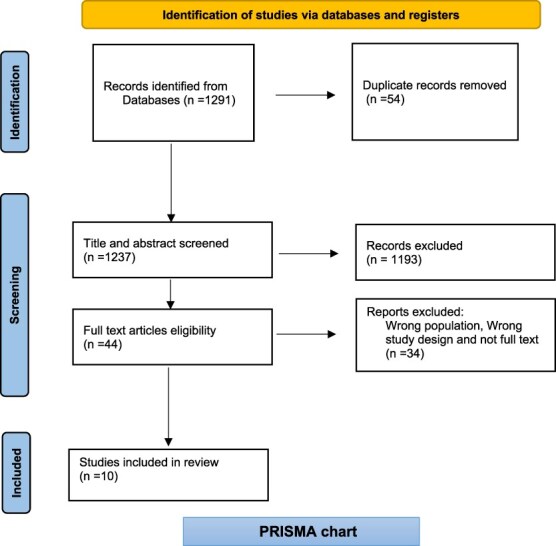
Flowchart showing the study selection process.

As depicted in Fig. 1, of the 44 full text articles assessed, 34 were excluded as follows:

- 12 due to wrong population (patients or non-health professionals rather than clinicians)
- 10 due to quantitative or mixed methods study designs
- 12 because full texts were inaccessible or not peer reviewed

### CASP

‘Critical Appraisal Skills Programme’ (CASP) is a tool that plays a vital role in the rigour, methodology, and quality of the study (Long et al. 2020). CASP provides a structured framework for the evaluation of study design, sampling methods, data collection, and analytical approaches (Renjith et al. 2021). The use of CASP tools enables researchers to assess the strengths and weaknesses of individual studies, thereby enhancing both the reliability and validity of their findings (Long et al. 2020). CASP has been incorporated into this review to meet high-quality standards and reinforce its credibility as shown in Table 2 (Renjith et al. 2021). Standardized questions are used to evaluate each study (Long et al. 2020).

**Table 2:** CASP table.

		Scores (numbered 1–10)										Total
S. No	Author and year of publication	1	2	3	4	5	6	7	8	9	10	
1.	Ganapathi and Duggal (2023)	Y	Y	Y	Y	Y	Y	Y	Y	Y	N	9
2.	Russell et al. (2023)	Y	Y	Y	Y	Y	Y	Y	Y	Y	C	9
3.	Fischer et al. (2023)	Y	Y	Y	Y	Y	Y	Y	Y	Y	C	9
4.	Nash et al. (2023)	Y	Y	Y	Y	Y	Y	Y	Y	Y	N	9
5.	Barry et al. (2022)	Y	Y	Y	Y	Y	Y	Y	Y	C	N	8
6.	Held et al. (2022)	Y	Y	Y	Y	Y	Y	Y	Y	Y	N	9
7.	Huang et al. (2023)	Y	Y	Y	Y	Y	Y	Y	Y	Y	C	9
8.	Wang et al. (2021)	Y	Y	Y	Y	Y	Y	Y	Y	Y	C	9
9.	Haugsten et al. (2023)	Y	Y	Y	Y	C	Y	Y	Y	Y	C	8
10.	Samhammer et al. (2022)	Y	Y	Y	Y	Y	Y	Y	Y	C	C	8

### Data extraction

Articles were obtained using the selection criteria (Butcher et al. 2020). After duplicates were removed, titles and abstracts were screened (Butcher et al. 2020). Full-text articles were then evaluated to ensure they met the research objectives (Boren and Moxley 2015). Qualitative studies focusing on healthcare professionals’ perspectives on the utilization of AI tools, as well as those detailing facilitators and challenges of AI tool use in clinical settings, were included. Studies employing quantitative methods, mixed methods, or exploring patient opinions were excluded.

Ten articles reporting healthcare professionals’ perspectives from India, the UK, Canada, Singapore, China, and the USA were selected to capture insights from both developed and developing nations as shown in Table 3. Studies from rural areas and primary care settings were also included to identify context-specific challenges.

**Table 3:** List of included studies.

Author and publication year	Research design and sample size	Aim of the research	Key findings
Ganapathi & Duggal (2023)	Semi-structured interviews with 11 English doctors	To explore NHS health professionals’ experiences of working with AI, its implementation and future engagement	Health providers are interested in collaborating with AI and considering it as a career option, but there is a lack of organized pathways and a multidisciplinary approach. Frontline doctors have limited awareness and lack sufficient skills to operate. Healthcare professionals should be engaged in developing AI tools.
Barry et al. (2022)	Semi-structured interviews with 29 health professionals	To explore primary care providers’ perspectives on an AI-based screening tool for identifying heart failure	Health professionals understood the clinical value of the tool for diagnosis; training is essential. Establishment of guidelines incorporating clinical reasoning and effective resource allocation is required. Workflow adaptations and strengthened physician–patient interaction are also necessary.
Held et al. (2022)	Semi-structured interviews with 24 participants	To explore healthcare providers’ perspectives on facilitators and challenges of an AI-supported screening tool for diabetic retinopathy in primary care	Providers expressed a positive attitude and highlighted the need for effective practice strategies, organizational responsibilities, training, and technical support. Factors such as finance, professional impact, patient safety, and validity were prioritized.
Fischer et al. (2023)	Semi-structured interviews with 13 participants	To explore obstetrics healthcare workers’ perspectives on AI usage and its role in the clinical setting	Clinician trust requires validity, explainability, and positive individual experience. Clinicians expect AI to extend beyond human capabilities. AI must consider contextual factors and incorporate new parameters to ease clinicians’ workload.
Nash et al. (2023)	Semi-structured interviews with 27 participants	To examine primary care professionals’ experiences of using AI tools	Health professionals have limited knowledge about AI. Trust is built through accuracy and transparent integration. Internal influences include efficiency and decision-support; external influences encompass privacy protection, liability, and finance. Concerns also arose regarding clinician–patient interaction.
Huang et al. (2023)	Semi-structured in-depth interviews with 30 Indian and 15 Singaporean physicians	To explore clinicians’ perspectives in India and Singapore on an AI-based CDSS for prescribing antibiotics	Singaporean clinicians were favourable towards an AI-supported prescribing tool, whereas Indian clinicians were sceptical. Both groups expressed concern over over-reliance. Essential requirements included workflow integration, validated algorithms, comprehensive training, technical support, and cost-efficiency.
Wang et al. (2021)	Semi-structured interviews with 22 participants	To understand clinicians’ perceptions of ‘Brilliant Doctor’, an AI CDSS in rural clinics, and its challenges in clinical practice	Clinicians viewed the CDSS positively, noting its assistance in diagnosis, identification of rare conditions, and prevention of adverse events. Challenges included technical support, usability, transparency, trust, clinician autonomy, rural organizational issues, and workflow integration.
Samhammer et al. (2022)	Semi-structured interviews with 14 participants	To understand nephrologists’ perceptions of the AI decision support system in clinical practice	Clinicians’ experiences and multidisciplinary perspectives were found to be critical in decision making. Concerns centred on AI transparency, explainability, and the impact on clinician–patient interaction.
Haugsten et al. (2023)	Focus-group interviews with 14 participants	To investigate health professionals’ experiences of the AI-supported ATBM Master (FotoFinder) tool for total-body dermoscopy in skin lesion diagnosis	Professionals highlighted the absence of AI usage guidelines and insufficient training. Reported benefits included reduced bias and comprehensive overview; drawbacks comprised low specificity, time pressure, and technical limitations. The tool was viewed as a supplement rather than a replacement for clinicians.
Russell et al. (2023)	Semi-structured interviews with 15 participants	To explore healthcare professionals’ competencies regarding the use of AI-based tools	Health workers agreed that AI tools support care but require greater understanding, skills, and continuous training with a patient-centred focus. Providers must prioritize social, legal, and ethical concerns for AI deployment. AI tools should integrate seamlessly into workflows, and community engagement is needed to reduce bias.

### Data analysis

Thematic synthesis was used for data analysis, as it is effective in elucidating complex qualitative information. Open coding was employed, which facilitates the translation of ideas across studies and generates new codes (Thomas and Harden 2008). Recurring and unique data were coded, and a hierarchy of codes was developed by comparing similarities and disparities (Thomas and Harden 2008). Descriptive themes were constructed using these codes, with thematic saturation appropriately maintained (Hogg et al. 2023). All themes were then interpreted and organized to meet the research objectives, including information on the initial themes (Thomas and Harden 2008).

## Results

From 10 selected qualitative studies, a variety of themes regarding the use of AI tools in clinical practice were identified. Six themes were developed to support the objectives of this research: behaviour; perceived usefulness; performance expectancy; ethical and legal aspects; challenges; and AI-tool proficiency. Verbatim are provided in Supplementary Appendix A.

### Behaviour

Healthcare professionals must develop a sound understanding of AI tools, including their components, algorithms, and capabilities, to ensure the safe delivery of patient care (Nash et al. 2023, Russell et al. 2023). In cases where AI-generated suggestions appear inconsistent, clinicians tend to rely on their own clinical judgement (Fischer et al. 2023). While many expressed enthusiasm to integrate AI due to fatigue with traditional methods (Ganapathi and Duggal 2023), others, particularly part-time practitioners, remain hesitant, valuing the credibility afforded by face-to-face practice (Ganapathi and Duggal 2023). Nonetheless, AI adoption has been associated with enhanced job satisfaction, reduced stress, and better work–life balance (Ganapathi and Duggal 2023).

### Perceived usefulness

Clinicians are increasingly open to integrating AI-supported technologies in healthcare (Barry et al. 2022, Held et al. 2022). Tools such as total body dermoscopy (TBD) offer unbiased monitoring of skin lesions (Haugsten et al. 2023), while AI-driven clinical decision support systems like ‘Brilliant Doctor’ assist diagnosis, education, and alerts, despite some reported limitations (Wang et al. 2021). In Singapore, AI improved antibiotic prescribing decisions, whereas Indian clinicians were more sceptical (Huang et al. 2023). AI has enhanced diabetic retinopathy screening and reduced workload via collaborative practices (Held et al. 2022), and it supports remote care through automated reminders (Held et al. 2022, Fischer et al. 2023). However, TBD tools can be time-intensive, and AI may miss contextual insights (Fischer et al. 2023, Haugsten et al. 2023, Nash et al. 2023).

### Performance expectancy

Although structured guidelines enhance trust, healthcare professionals advocate for tools that are both explainable and comprehensible, facilitating risk factor identification (Fischer et al. 2023). Explainability aids in evaluation, error detection, tool control, and effective interaction (Samhammer et al. 2022). Professionals further emphasized that AI should surpass human capacity in decision support (Fischer et al. 2023). Integration must also enhance workflow by improving multidisciplinary coordination (Russell et al. 2023). Efficient clinical systems require strong IT infrastructure, interoperability, accessible patient data (Ganapathi and Duggal 2023), sound organizational strategies, technical support (Held et al. 2022), algorithm updates, cost efficiency, and feedback mechanisms (Huang et al. 2023).

### Ethical and legal aspects

Standardized regulation is vital to resolve ethical and legal concerns in AI use (Russell et al. 2023). Transparent algorithms are necessary to gain trust and support interpretability, as AI often operates as a ‘black box’ (Samhammer et al. 2022, Ganapathi and Duggal 2023). Regular audits based on evidence and clear legislation on liability are essential (Fischer et al. 2023, Huang et al. 2023). Health professionals report insufficient guidance on AI tools, calling for dedicated practice guidelines to ensure scientific rigour (Fischer et al. 2023, Ganapathi and Duggal 2023, Haugsten et al. 2023). AI use should remain voluntary to protect clinician autonomy (Huang et al. 2023), prioritizing patient safety and data privacy (Held et al. 2022, Nash et al. 2023).

### Challenges

AI implementation in rural settings remains limited due to high patient volumes and time constraints (Wang et al. 2021). Challenges include inaccuracy, bias from under-representation, technical failures (Wang et al. 2021), insufficient multidisciplinary collaboration (Ganapathi and Duggal 2023), and financial limitations (Nash et al. 2023). Clinical trials are vital to ensure the scientific rigour and reliability of AI tools (Russell et al. 2023). Contextual variations are notable: in Singapore, shared AI-driven decisions are common, unlike in India, where financial pressures limit AI use (Huang et al. 2023). Weak physician–patient relationships persist where training is inadequate (Barry et al. 2022, Samhammer et al. 2022).

### Artificial intelligence-tool proficiency

Healthcare professionals recommended practical training in AI tools to build operating proficiency before clinical deployment, alongside ongoing technical support (Wang et al. 2021, Barry et al. 2022, Haugsten et al. 2023). Nevertheless, insecurities persist among clinicians regarding AI (Held et al. 2022). Introducing AI into undergraduate curricula can help graduates to pursue relevant career paths (Ganapathi and Duggal 2023). Involving health professionals in AI development was advised to yield more integrated outcomes (Ganapathi and Duggal 2023), alongside continuous professional development through practice-based education (Russell et al. 2023).

## Discussion

This systematic review seeks to examine healthcare professionals’ perceptions regarding the integration of AI-supported tools within routine clinical practice. The analysis identified six overarching themes: behaviour, perceived usefulness or performance expectancy, ethical and legal considerations, challenges, and proficiency in using AI tools.

Healthcare professionals acknowledged the necessity of possessing operational knowledge of AI tools, highlighting that such understanding fosters trust, mitigates uncertainties, and ensures the provision of safe and effective patient care (Russell et al. 2023). Despite a clear interest among clinicians in expanding their expertise in AI, there appears to be a notable absence of structured learning pathways to support this endeavour (Ganapathi and Duggal 2023). This observation is consistent with existing literature, which underscores the significance of equipping healthcare professionals with appropriate knowledge and competencies to ensure successful AI integration into clinical practice (Castagno and Khalifa 2020, Garvey et al. 2022, Catalina et al. 2023).

AI tools offer substantial support across diverse clinical domains, including diagnostic processes through clinical decision support systems (Wang et al. 2021), dermoscopy for skin lesion detection (Haugsten et al. 2023), antibiotic prescription (Huang et al. 2023), diabetic retinopathy diagnosis (Held et al. 2022), and time management improvements (Fischer et al. 2023). Additional studies confirm the utility of AI across specialisms such as cardiology, radiology, and pharmaceutical practices, and in emergency settings (Choudhury and Asan 2020, Alanazi 2023, Bajgain et al. 2023).

The development of a robust legal framework is essential to safeguard patient data, uphold governance, and define accountability in AI utilization (Huang et al. 2023), Fischer et al. 2023). Furthermore, there is a call for the establishment of clinical guidelines that align with standard medical practices and maintain scientific integrity (Fischer et al. 2023, Ganapathi and Duggal 2023). Many healthcare workers advocate for AI tools to be used voluntarily, safeguarding their professional autonomy (Huang et al. 2023), a view reflected across literature which prioritizes ethical, legal, and social dimensions alongside clinician independence (Gerke et al. 2020, McGreevey et al. 2020, Rodrigues 2020, Cartolovni et al. 2022, Naik et al. 2022, Siala and Wang, 2022, Bhavaraju 2023).

Transparency in AI algorithm design is considered vital for fostering trust, particularly when clinicians’ own judgement contradicts AI-generated suggestions (Samhammer et al. 2022, Fischer et al. 2023). This aligns with recommendations promoting explainability in AI to facilitate clinician comprehension (Cath 2018, Amann et al. 2020, Ghassemi et al. 2021). Moreover, AI tools should incorporate clinical reasoning, contextual awareness, continual updates, and affordability, considering patient-specific financial constraints (Emani et al. 2022, Fischer et al. 2023, Huang et al. 2023). Although many clinicians in the included studies emphasized explainability, other scholars argue its necessity remains debated (Ghassemi et al. 2021). This synthesis suggests that while not all clinicians demand full algorithmic transparency, they consistently value explanations that enhance error detection, workflow integration, and patient communication.

Clinicians stressed the need for AI to support workflows via resilient Information Technology (IT) infrastructure, technical support, interdisciplinary collaboration, and strategic implementation (Aung et al. 2021, Blezek et al. 2021, Held et al. 2022, Russell et al. 2023). Nonetheless, challenges persist, including technical malfunctions, underrepresentation of diverse populations in datasets (Wang et al. 2021), insufficient trial-based evidence (Russell et al. 2023), and potential disruptions to patient–clinician relationships (Huang et al. 2023). These concerns are echoed in research highlighting the risks posed by poorly representative data (Wahl et al. 2018, Liaw and Kakadiaris 2020, Petkus et al. 2020), the predominance of preclinical studies (Reddy et al. 2019, Sun and Medaglia 2019), and the importance of sound engineering practices to prevent postimplementation failures (Rong et al. 2020).

Finally, healthcare professionals recommended embedding AI training into educational curricula and emphasized the importance of incorporating clinicians’ perspectives into AI tool development (Barry et al. 2022, Ganapathi and Duggal 2023). This perspective is supported by literature calling for clinician involvement in AI design (Ross et al. 2016, Hashimoto et al. 2018), skill enhancement through structured training (Lebcir et al. 2021, Wiljer et al. 2021), and improved AI literacy to elevate patient outcomes (Laupichler et al. 2022).

### Limitations

This systematic review primarily concentrates on healthcare professionals’ perspectives; it is essential to include views of multiple stakeholders, including patients and developers. Quantitative studies were not included, limiting the breadth of findings.

## Conclusion

The review studies indicate that AI tools can support safer care by enhancing diagnostic accuracy (Barry et al. 2022, Held et al. 2022) and improving workflow efficiency (Ganapathi and Duggal 2023). Some clinicians also perceived potential cost savings through resource optimization (Huang et al. 2023). However, these benefits depend on proper training, workflow integration, and ethical safeguards. Healthcare professionals recognize the importance of AI technology and are prepared to integrate it to improve care. However, they require competencies for effective AI tool usage and must be involved in AI development to meet clinical needs. It is essential to prioritize ethical and legal aspects—such as autonomy, transparency and accountability—and to establish guidelines that foster clinician trust. AI tools should promote efficient workflows through robust infrastructure and strengthen patient–clinician relationships. Incorporating explainability, improving algorithmic accuracy, and enacting legislation for secure data use are critical for the safe utilization of AI in healthcare. The review reinforces that while AI tools may reduce errors and support decision making, physicians’ good clinical practice skills, accountability, and patient centred judgement remain the foundation of care. AI is an adjunct, not a substitute, and trust in the medical profession relies on this distinction.

## Supplementary Material

oqag004_Appendix

## Data Availability

The data underlying this article are derived from previously published studies, which are cited in the reference list. No new primary data were generated or analysed for this systematic review.

## Data sharing statement

All data supporting the findings of this review are presented within the main text and in the Supplementary Material.
