# Supplementary material for: Healthcare professionals’ perspectives on artificial intelligence in clinical practice: a systematic review of facilitators and challenges
Source: Oxf Open Digit Health. 2026 Feb 10;4:oqag004. doi: 10.1093/oodh/oqag004 (PMC12935013; doi:10.1093/oodh/oqag004)
Supplement: oqag004_Appendix [file oqag004_appendix.docx]

Healthcare Professionals’ Perspectives on Artificial Intelligence in Clinical Practice: A Systematic Review of Facilitators and Challenges

**Appendix A**

| **Author** | **VERBATIM** | **THEME** |
| --- | --- | --- |
|  |  |  |
| Russell et al. 2023) | “The problem there is that the people that  are using those tools don’t know about  the individual studies that went into the  data underlying the tool. There may have  been exclusion criteria for, like, people  under 70 weren’t admitted into the trial,  or children weren’t admitted into the  trial, and so on. And that may not have  been true of all of the trials, but enough  of them that it would bias the underlying  statistical basis for predictions for outliers.  So, it’s very dangerous to apply a tool, in  general AI tools, if you don’t know what  their boundary conditions are”. | Concerns about bias |
| Fischer et al. (2023) | “If you know what that model is based on, it is not some mysterious black box where something comes out, but we as doctors know what those models are based on and what parameters are included. Then I  can live with it”. | Explainability |
| Nash et al. (2023) | “And I do not know enough about artificial intelligence to give you big ideas of what could be done.” | Healthcare professional knowledge |
| Samhammer et al. (2022) | “the patient, I, other disciplines, laboratory physician and radiology and a senior physician. And I think sometimes, of course, the other team, other assistants, who point out something, who maybe did the examination, the nursing, I don’t know, the patient tells the nursing staff  something else again or they notice something again, which I didn’t notice. So there are more people”. | Interpersonal exchange |
| Huang et al. (2023) | “I guess at the end of the day, if you don’t make it a hard stop, the responsibility is in the clinicians because we can choose to follow, or we can choose to override.” | Supportive medico-legal framework |
| Barry et al. (2022) | “You can tell them, thankfully, it’s normal. The EKG picked up something that showed potential for concern. We have good news that everything is  normal. We’re gonna continue to optimize your treatment. That being said, it’s several thousand dollars”. | Cost- effectiveness and Resource Allocation |
| Ganapathi and Duggal (2023) | “I now work predictable hours, I have time to go to the toilet when I want to, I have time to eat my lunch when I want to, I have time for my family and friends and I do not feel anxious at my work as well” | The route to working with AI |
